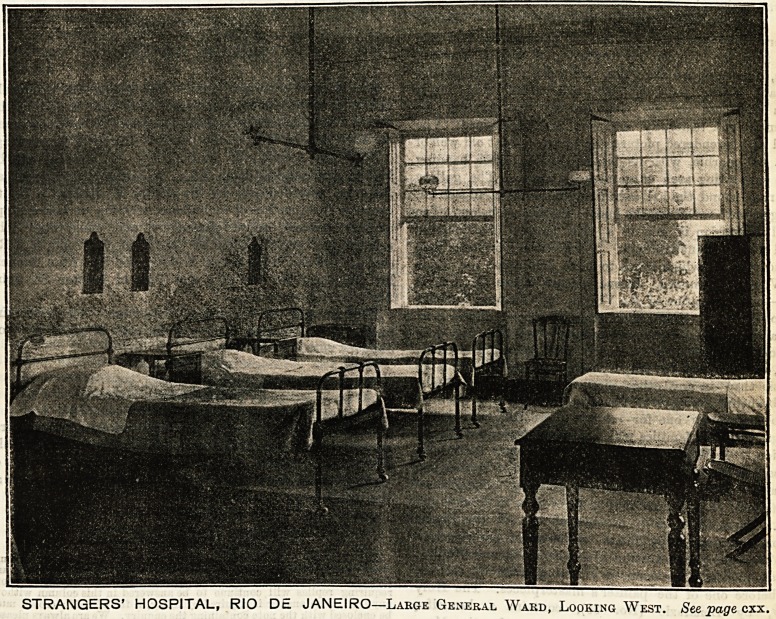# The Hospital Nursing Supplement

**Published:** 1895-01-19

**Authors:** 


"The Hospital" Nursing Mirror.

Being the Extra Nursing Supplement of "The Hospital" Newspaper.

[Contributions for this Supplement should be addressed to the Editor, The Hospital, 428, Strand, London, W.C., and should have the word "Nursing" plainly written in left-hand top corner of the envelope.]

### News from the Nursing World.

### METROPOLITAN ASYLUMS BOARD NURSES.

The recommendations which the Nursing Staff Committee recently made to the Metropolitan Asylums Board with regard to the time-off-duty of their nurses have been adopted, and monthly leave now consists of a day, which commences at seven a.m. The Superintendent and the charge nurses are allowed to sleep out of the hospital on the night preceding their day off duty, of course with the permission of the medical superintendent and matron. Extra passes may be granted from half-past eight p.m. to ten, "when convenient to the administration." The advantage of the first rule is obvious, as it enables day nurses to start fresh for their holiday without doing any previous ward duty. Permission to sleep away from the hospital is also a highly-prized addition to a day's leave, but the benefit of the passes given from half-past eight p.m. to ten is not so easy to recognise. The elaborate personal disinfection and changing of clothes imperative on nurses in infectious hospitals every time they go out are serious undertakings at an hour when weariness and conscientiousness will assuredly oppose each other. For the junior members of the staff there are also many drawbacks to using such extra leave in neighbourhoods where some of the Board's hospitals are situated, although in others no doubt evening outings may be safely indulged in.

### THE TEMPERANCE HOSPITAL.

To a busy, noisy thoroughfare like Hampstead Road, a pleasant contrast is presented by the pretty wards of the Temperance Hotel, where the patients always have a well cared-for look, and their surroundings are bright and peaceful whatever the weather without. Moreover, kindliness and courtesy appear to pervade the institution, no visitor desirous of seeing modern appliances or new ward furniture need hesitate to ask for the necessary permission to view them at this attractive little hospital.

### LITTLE INVALIDS.

A concert is announced for the afternoon of January 22nd, when an excellent programme will be carried out by a number of artistes who have kindly offered their services, to help the funds of the Invalid Children's Aid Association. This society ought to be widely known and supported, for its work is unique and valuable. It provides visitors to and confers substantial benefits on the children discharged from hospitals or unsuited for admittance thereto. The association also pays for long visits to convalescent homes or for surgical appliances, and it lends invalid carriages and chairs to frail little creatures, who could never otherwise be taken out of doors. In fact, the usefulness of the I.C.A.A. is unquestioned, and this concert, which is under the patronage of H.R.H. the Duchess of York, should give it some of the financial support which it needs.

### ANNUAL TAXATION.

In pleasing contrast to the prevalence of presentations from nursing staffs to superintendents comes the story of a well-known matron, who in her own probationer days saw some evils in the custom, and, on her promotion to office, waged war against it. She knew the annual collections for birthday and Christmas gifts formed a heavy tax on many purses, whose owners shrank from confessing that they could not afford the luxury of giving. The new matron therefore let it be understood in the school that she not only disapproved of so-called presentations, but that she should absolutely decline ever to accept any such outward and visible signs of esteem and gratitude; by this act she probably secured a large measure of both!

### A NURSES' HOME WANTED.

A new home for the nurses is being agitated for at the Sussex County Hospital, Brighton, and funds are urgently required for the purpose, the present accommodation being inadequate. The proposed structure will not only add to the personal comfort of the nurses, but will also, by setting free the rooms occupied by them in the old building, render increased space available for the out-patients and officials.

### TRAINED NURSES AT DUNDEE.

At a meeting held last week at the Caird Home of Nurses at Dundee, most cordial acknowledgment was made as to the value of trained district nurses. The speakers testified to the good influence which had been brought to bear by the Queen's nurses on all with whom they came in contact, and also to "the cheerful, orderly, and kindly manner" in which arduous duties were performed. Certificates granted by Queen Victoria's Jubilee Institute were handed to Nurses M'Dougall, M'Kensie, and Mitchell, by Mr. Moncour, the president, who stated that to obtain them, not only length but efficiency of service was essential. The presence of numerous ladies and gentlemen proved that much local interest is taken in the Dundee Branch of the Q.V.J.I.

### PRIMITIVE DRESSINGS AND DIETS.

The presence of trained nurses in workhouses is sometimes objected to, not only by guardians and masters, but by certain of the sick paupers, who are inclined to oppose the skilful application of dressings to bad legs. Hitherto ointment and linen distributed to the patients for this purpose have sometimes been kept in their lockers, to be applied at their own discretion. A wound, painless so long as the limb was at rest, was naturally valued by its owner as a protection against a return to the active work of the institution. Dressings applied regularly by a trained nurse, therefore, heal up the legs much too fast to suit the taste of the pauper, who looks with wrath upon innovations, reflecting that "if a man's legs ain't 'is own to treat as he likes, things is come to a pretty pass." The lockers may be also the receptacles of the whole day's allowance of food, and the wardsmen and women being also liberally dieted, it has been proverbially easy for typhoids and other cases to obtain substantial additions to liquid food ordered by the doctor, and which by the patient is considered a form of "just starvin' the pore fellow outright!" Tact as well as training are evidently demanded of the new workhouse nurse.

### AMERICAN WOMEN AS PROFESSORS.

The Professor's Chair of Visceral and Histological Anatomy at the Kansas City College of Physicians and Surgeons is now occupied by a lady named Dr. Katherine Berry Richardson. At the University of Michigan it is reported that, by a recent decision of the Board, women are now eligible for professorships; and at Detroit a proposal has been made to establish and endow a special female professorship. For the latter purpose nearly half the sum required has been subscribed.

### WITHOUT NAMES.

Why are nurses ashamed of their names? Possibly an indignant denial will meet this suggestion, for the ladies figuring, say, as "Sister Jane" or "Sister Amelia" tell us that modesty or their family's feelings necessitate this abolition of surnames. Of course, we are not referring to religious orders whose members are under obligation to take names to which they are often neither born nor baptised. It is the secular nurse who frequently figures as "Sister" in provincial and metropolitan journals, whilst even untrained women undertaking to teach in lectures the art of nursing constantly pose as professional nurses. Nameless speakers are equivalent to anonymous correspondents in the eyes of those who consider that a full signature is the best guarantee of good faith.

### REGISTRATION OF MIDWIVES.

Under the presidency of Sir F. Fitzwygram, M.P., a meeting was held on the 9th inst. at the Midwives' Institute, 12, Buckingham Street, London, to consider the expediency of the promotion of a Bill for the registration of midwives. A discussion took place as to whether a Royal Charter would in this case be preferable to an Act of Parliament, but the opinion of most of those present was favourable to the latter. The object of a Bill, the chairman explained, was to secure a better position for midwives, and to protect mothers from those who were incompetent and unqualified. It was decided to form a "Parliamentary Bills Committee," which should consist of a member from each of the following societies: The Obstetrical, the Gynæcological, the Midwives' Registration Association, the North of England O. and G. Association, the British Medical Association, the training schools of the four portions of the United Kingdom, two Members of Parliament, one representative from the Midwives' Institute, and one gentleman practitioner; Dr. Rowland Humphreys being appointed secretary. Miss Hampson, Matron of the Rotunda Hospital, was one of the speakers at the meeting, at which a large number of ladies and gentlemen were present, including Dr. Schwann, M.P., Dr. Potter, Dr. Rayner, Dr. Leith Napier, Dr. Watt Black, Dr. Boxall, and Dr. Herman. Letters of regret for unavoidable absence and of sympathy with the meeting were read from Sir John Williams, Bart., Dr. Danford Thomas, Dr. Playfair, Dr. Collins, Dr. Percy Boulton, Dr. Thomas Wilson, Dr. Holland, Mr. H. Fell-Pease, M.P., and others.

### WHO DOES THE WORK?

Needlework guilds appear increasingly popular, and might be attached to every hospital, thereby ensuring a steady supply of suitable garments for convalescent and other patients. The response to our own annual appeal is particularly gratifying on account of the testimony borne by it to the self-denial of our nurse readers. Materials and time are precious offerings, and these are freely given by the contributors to our Christmas parcels. Highly do we appreciate the gifts which they put it in our power to send to many hospitals. We could wish that more outside interest were shown in this matter. Many employers of private nurses, convalescent, well-to-do patients, might easily accomplish something for their less fortunate neighbours. Next year we hope that in addition to the parcels which are sent by nurses and other busy workers, more offerings may come from those who have leisure and means to give substantial aid in the matter.

### "THE HOSPITAL" CONVALESCENT FUND.

From Miss E. B. Gray a kind donation of twenty shillings has been received for this fund, which we have much pleasure in acknowledging.

### INFIRMARY NURSING.

The Workhouse Infirmary Nursing Association is to be congratulated on the year's progress, especially on having supplied nurses to many new Boards, including Ampthill, Bridgend, Cowbridge, Cosford, Headington, Lichfield, Malmesbury, Plymouth, Spalding, St. Colomb, Major, Sherborne, and Weymouth. The recommendations with regard to the better payment of nurses have also been followed by several Boards of Guardians, and the patient, steady work done by the association in this and other respects has done much to advance the cause for which it exists.

### SHORT ITEMS.

The matron of the Chelsea Hospital for Women has sent in her resignation, and so have various members of the nursing and domestic staffs.—An "At Home" given by the superintendent of the London Association of Nurses on the 8th inst. was well attended by members, to whom a very pleasant evening's entertainment was given.—A successful report of the Darwen Nursing Association was presented at the recent annual meeting.—The Barton Regis Board of Guardians have decided to add two trained nurses to the workhouse infirmary staff.—The sum of £23 6s. 7d. was realised for the Queen Victoria Nursing Institute at Cardiff by a recent musical and dramatic entertainment.—Mme. Brière (Sister Elise) has been nominated a Chevalier de la Légion d'Honneur in acknowledgment of her devoted service to the lepers at Guadaloupe as "Superior" of their hospital, La Désirade.—Miss Honnor Morten has been selected from a large number of applicants to give a course of lectures on "Ambulance and Hygiene" under the London County Council.—At the general annual meeting of subscribers to the Newbury Parish Nurse Fund it was stated that 160 articles of clothing had been contributed by the Children's Needlework Guild.—A well-attended meeting has been held at Holyhead, at which it was resolved to secure a trained district nurse for the town; in an interesting address Miss Ingram, herself a fully-trained nurse and health lecturer, described the work undertaken by the Queen's nurses.—The verdict at the inquest on Nurse Emily Susan Marks, who unfortunately died on 5th inst., stated that her death was due to an overdose of morphia, inadvertently injected by herself.

### Elementary Anatomy and Surgery for Nurses.

By W. McAdam Eccles, M.B., B.S., F.R.C.S., Lecturer to Nurses, West London Hospital.

### II.—THE OSSEOUS SYSTEM.

In this lecture the bony framework of the body will be dealt with. At the outset we are met with the question, what are the chief uses of a hard compact tissue like bone distributed throughout the body? To this query three replies may be given: (1) That the bones serve as a support to the soft parts; (2) that they act in certain situations as a protection to important organs; and (3) that they provide firm attachments for muscles to act from and upon.

The different bones of the body can to a great extent be well studied by a careful examination of the skeleton of one

Fig. 1.—The Skeleton.

of the lower vertebrata, for instance that of a dog or rabbit, and if an articulated human skeleton be not within reach it is highly desirable that one of the above should be used.

The adult human subject has no less than 200 separate bones entering into the osseous system, which may be grouped as follows: (1) The skull, including the face, 22; (2) the vertebral column, 26; (3) the hyoid bone, 1; (4) the sternum and ribs, 25; (5) the upper extremities, 64; (6) the lower extremities, 62. These, again, from their respective shapes, fall into four divisions: (1) Long bones, *e.g.*, the humerus; (2) short bones (including sesamoid bones), *e.g.*, the wrist bones; (3) flat bones, chiefly those of the vault of the skull; (4) irregular bones, *e.g.*, the vertebræ.

It will thus be seen that there is a great diversity among the component parts of the skeleton, and this no doubt to allow of their perfect fitness, which they as individual bones require to fulfil their several functions, and when grouped to act in co-ordination. The bones must therefore one and all be reviewed separately in order to thoroughly master their general form and principal features; but beyond this they must be carefully studied in their relationship to one another.

### The Skull.

1. *The Bones of the Skull.*—Two divisions here have to be recognised, viz., the bones constituting the cranium proper and those forming the face. If in a skeleton the skull be looked at it will be seen that there is a large cavity entirely surrounded by bone, save for certain apertures. This is the cranium, the space within which the brain itself is lodged, and the bone affords a very efficient protection to the delicate nervous tissue. Attached to this cranium will be observed a number of irregular bones, which constitute the face; but these are little developed in quite a young child.

(*a*) There are eight bones, which are intimately connected with each other, entering into the composition of the cranium —the occipital, the two parietal, the frontal, the two temporal, the sphenoid, the ethmoid. The whole of the parietals, and part of the occipital, the frontal, and the temporals form the vault of the skull, while the whole of the sphenoid, and the ethmoid, and part of the occipital and the temporal enter into the base of the skull.

The occipital bone presents a somewhat triangular tabular portion, which externally is smooth above, entering into the vault of the skull, but below has ridges and depressions for the attachment of muscles, whose functions are the production of certain movements of the head. A well-marked prominence on this surface—the external protuberance—can be felt beneath the skin. Internally there are four fossæ or pits which lodge parts of the brain; they are separated from each other by marked ridges, which themselves are grooved. One of the most interesting features to notice in the occipital bone is the large aperture—the foramen magnum—which exists in its under surface. It is through this that the spinal cord becomes continuous with the brain. On either side of this on the under surface of the bone will be seen two convex projections, termed the condyles. It is by means of these that the occipital bone articulates, or becomes connected with, the first of the vertebræ, and it is between these two bones that the to and fro, or nodding, movements of the head take place. Besides this the occipital touches both the parietal and both the temporal, and in front in the adult is firmly joined by osseous tissue with the sphenoid, though in the child the two bones are quite separate.

The parietal bones are symmetrical, and are roughly square in shape, smooth except for a slight ridge, and convex externally, but marked by depressions and grooves, and concave internally. One of these grooves at the lower and anterior angle is usually very deep, sometimes a perfect canal in the bone, and is occupied by an artery which may be torn if the parietal bone happens to be fractured. Each parietal forms the great part of the side and roof of the cranium, and articulates with its fellow of the opposite side, with the occipital, with the temporal of its own side, with the frontal, and with the sphenoid.

The frontal bone forms the forehead. In the child it consists of two symmetrical halves, which in the adult become fused. Besides the vertical part entering into the vault of the skull there is a horizontal portion, deeply cleft, each half of which forms part of the roof of the corresponding orbit or socket for the eye, as well as part of the roof of the nose. The frontal articulates with twelve bones—the two parietal, the sphenoid, the ethmoid, the two nasal, the two superior maxillary, the two lacrymal, and the two malar.

The temporal bones are again alike on the two sides. Each has a flat portion which forms part of the side wall of the cranium, and from which projects a slender process which completes an arch, easily felt, with the malar or cheek bone. Besides the portion entering into the vault of the skull, there is a strong pyramidal part in a horizontal plane which forms part of the base of the skull. A well-marked prominence—the mastoid process—which is developed in the adult, but absent in children, can be plainly felt behind the ear. On the lower surface of the bone is a sharp spine, often broken off in the skeleton, called the styloid process, which serves for the attachment of muscles. On the under surface also is a deep pit—the glenoid, or socket fossa—for the reception of the condyle of the lower jaw. The temporal bone encloses the organ of hearing, and presents an aperture externally for the entrance of sounds, which is termed the external auditory meatus. This bone articulates with five others—the occipital, the parietal, the sphenoid, and, lastly, the inferior maxilla.

The sphenoid bone is shaped something like a bat, having wings and processes representing feet. It is wedged in between the other bones at the base of the skull.

The ethmoid bone is situated in the cleft between the two parts of the horizontal portion of the frontal bone. It is spongy in character, and perforated by numerous foramina like a sieve, hence its name.

### The Madhouse of Fiction.

We wonder how many of those who read the stories issued as supplements to popular weekly papers believe that they represent phases of life which are in any way true or possible. We take up a paper which, if we are to credit the title-page, exists to elevate and amuse, and yet its descriptions equal the most transparent absurdities ever invented for a comic journal. If novelists consult a lawyer to keep them right in matters of law, surely writers of tales in which asylums figure should take some precautions lest their ignorance becomes too ridiculous. From the style of this story and its contradictory statements, we should not perhaps expect such forethought from the author, but an editor ought to know enough to prevent the admission of such rubbish to his pages. From internal evidence we judge that the incidents narrated are supposed to have occurred quite recently. The asylum described is situated, of all places, in Scotland. This is hard on the Scots, who consider that in the treatment of the insane they set an example to the whole world. A young woman, beauteous and much sinned against, is incarcerated there, and, we think, on good grounds. She affirms that she is the Virgin Mary, and goes off into maniacal outbursts whenever she sees her husband. We give the author's own description of her: "There we were in the padded room. It was a terrible scene. I wished I had not accepted the Doctor's offer. That grandly beautiful creature lay on the floor, her clothes torn almost to rags, writhing in the folds of a strait waistcoat, and giving utterance to piercing shriek after shriek. Her long hair, beautiful still, swept loose over her face and neck, and every now and then she shook it back with the movement of an angry lion." Surely this conduct justified her detention. But it was all a piece of magnificent acting in order to escape from her husband because he got drunk on his wedding day. The proprietor of the establishment seemed to look upon her as one of the sights of the place, and invited a stranger to have a look at her.

Of course there is a detective mystery. The wily tracker of criminals gets himself, with the utmost ease, introduced into the asylum as a patient. This we may consider a stroke of genius, but it is nothing to what follows. He is admitted as a woman to the female side of the house! He takes with him a portmanteau with a lock constructed on a principle known only to two other men in Europe besides himself. Among other little necessaries this contains a suit of man's clothing, a dark lantern, a pair of shoes with list soles, and a bunch of skeleton keys. After this, we are not astonished to hear that he took with him a revolver and a strong, serviceable knife. He, or perhaps we ought to say she, was allowed to wander over the asylum and grounds on the day after his arrival. He learns from a patient, who believes herself to be Ophelia, that there is actually a padded room. This place can only be mentioned with a shudder. More than that, there is a cell in which a woman known as "the Bedlamite" has been confined for twenty years. There is no window to this den. She spends her life in total darkness, chained to the wall. As might be expected, the Bedlamite dies and is buried under the name of the heroine, while that martyr takes her place. Then we know what is going to happen. The detective, who must have had a supply of matches in his portmanteau, goes on a midnight prowl with his revolver and dark lantern. He surprises the hag of a matron in her bed, gags her, and binds her to the bedpost. He ransacks her papers, and finds a pathetic statement written by the heroine. His righteous indignation, stirred up by a perusal of her wrongs, is rapidly becoming too much for him, when—he hears the clank of a chain in the Bedlamite's cell. Quick as lightning, he snatches up his dark lantern, turns the light full on, and bounds thither. We will let him describe what he saw: "There, on the straw, which alone protected her from the cold stone floor; there, in that dark and noisome dungeon to which a ray of daylight never penetrated; there, covered with rags, and chained and padlocked to the wall like a wild beast, lay the once proud and beautiful wife of Sir Arthur Redleigh." To unlock her manacles is the work of an instant. To deal the hag of a matron (who is so inconsiderate as to yell for help) a blow with his fist which keeps her silent for hours, takes up only another moment. Then, with a cocked revolver in his right hand, the helpless heroine tucked under his left arm, and the lantern slung on to a button of his coat, he is ready for the fray. Nor was the enemy wanting. The doctor with *his* revolver, and backed by two burly ruffians armed with formidable bludgeons, bars the way. (Oh, Mr. Author, why did you forget the mastiffs?) At first the combat is but a wordy one, though the detective's "sharp menacing accents ring out like the rattle of musketry." Later on we have more serious business. The hero fires three shots with "deadly" effect, but apparently without seriously injuring anybody. He, however, bears off the heroine in triumph.

Can we be surprised that extraordinary ideas about asylums are current when we find the editor of an "elevating" paper publishing such nonsense? Does he really think it possible that a man could be admitted into the female wards of an asylum in the way described? Has he been sleeping for the last fifty years, that he fancies lunatics can be chained up in the dark for twenty years? Those who attend upon the insane can pass over the silly caricatures with contemptuous pity, but they must feel sorry for the editor, because on the page preceding the story is found in large type the words "If you see it in *Pearson's Weekly*, it's so."

### The Glasgow Western Infirmary.

### ADDRESS TO THE NURSES AT THEIR ANNUAL MEETING ON DECEMBER 29TH, 1894.

By JAMES FINLAYSON, M.D., Physician to the Glasgow Western Infirmary and to the Royal Hospital for Sick Children, Hon. Librarian to the Faculty of Physicians and Surgeons, Glasgow.

THE Western Infirmary aims not merely at treating the individual sick persons actually present in the wards, but at educating those who may have charge of the wards hereafter. The education and training of nurses has been kept in view since the beginning of the infirmary. Many of our nurses are now scattered over the country, some, indeed, in remote parts of the world—in Egypt, Australia, and elsewhere. In this country many of them occupy important positions as heads of institutions for the sick.

When this course of training was first started in 1875, those of us who were the responsible advisers of Miss Clyde and the directors in this matter were unanimously agreed that nothing less than a period of three years could be entertained as adequate. We felt sure that three years were actually required, for the educative influence of time is demanded for successful practical training in the wards. This was our first point. The next was to illumine the practical experience acquired in the wards by an intelligent conception of the rationale of what she was seeing and doing. This was secured by demonstrations and questioning, and cases were apportioned to be reported by the nurses. The last point was as regards the examination before the certificates were granted. These had to be occasionally refused or postponed as the case might be; but, however disagreeable for all concerned, this had sometimes to be done, or the affair would have degenerated into a form or a farce.

Now, after twenty years' experience, you may ask, What is the result? I do not think I am unduly lax; I confess I do not expect every nurse to be a paragon of perfection, or each individual to embody in her own person every virtue under heaven, but I think, on the whole, that our nurses are well trained, and that they are efficient. Many of you are no doubt aware that in various ways and under different phases an attempt is being made to change the training of nurses throughout the country, so as to secure uniformity in their training, and to stamp this uniformity by a system of national registration, somewhat on the model of our Medical Council and our Medical Register. In my opinion this is not an advance, but really a retrograde movement. If the honest truth were told our present medical system is far from satisfactory. We are constantly being told that the standards of the different bodies granting qualifications are far from being uniform or even from securing the necessary minimum. If this is so with the examination of medical students, how can we expect the examination of nurses, if conducted on a practical basis, and over a wide range of subjects, to have any great uniformity, even if the examiners were appointed by the same board, or even if all the nurses had to appear at one examination centre in London? It was not by assimilating our methods to those current elsewhere that we have succeeded in turning out the nurses we have done. If other schools, by more stringent preliminary examinations in sifting the raw material, which is now more abundant than formerly, or by more elaborate scientific courses of study, or by more rigorous educational discipline in the wards, can turn out, not, please observe, more learned but more useful nurses, then by all means let them do so, and let them have the credit of doing so; and let their nurses be run after for public or private work; and let us learn from them to amend our plans, and to profit by their experience. But in the meantime let all the training schools have a free hand, and work out their own distinctive methods as best they can.

With regard to registration, some nurses may, no doubt, feel flattered by the idea of an incorporation or a guild recognised by the Government of the country, but the Medical Register which it is proposed to imitate seems to me far from a satisfactory document. It is maintained at an enormous expense, yet scarcely a half-yearly session of our Medical Council passes without costly inquiries being instituted about removing certain names for misconduct of some kind; and law suits, expensive to both parties, are sometimes raised to have the deposed names reproved. A nurse's good name is as precious to her as a doctor's is to him. Are we to have all this costly inquiry concerning the register of thousands of nurses moving about, as they properly do, all over the country? If a strict watch is not kept over them, what is the security of registration? What guarantee does the fact of registration give for the continuance of the most ordinary moralities of life? How is a wandering body of nurses to be supervised? How is the register to be purged? If the project of uniformity of training and examination is illusory, the guarantee of a national register is a snare, if, indeed, the public are so stupid as to trust to its security. The certificates given to you here deal with the present; your own conduct is your only security for the future. That the nurses of the Western Infirmary may continue to uphold the reputation of their training school is not only my own earnest wish, but equally, I believe, the wish of all my colleagues, and of the directors and officials of this institution.

Before I conclude, I may say that I am far from thinking our plans perfect, or in any sense complete. I venture to speak of one point, and one point only, as my experience is that by keeping to one at a time we may have more chance of success. I refer to the instruction for our nurses in the preparation of food for the sick. This again cannot be taught properly without some consideration of the general subject of cookery. It is being more and more recognised that the subject of dietetics is of prime importance in the successful practice of medicine. But while our students are taught to amputate at the hip-joint, and to prescribe for every disease under the sun, how many of them can peptonise milk? Some of my nurses know what sort of concoctions have been turned out by some of our foremost students when set to do this, yet these are the men who have to instruct highly educated, or grossly illiterate mothers, as the case may be, on such points, when the life of a baby is trembling in the balance, or a patient may be fighting for his life at the height of typhoid fever. If this matter is important for our students, how much more for our nurses? still, the amount of real education in the preparation of food which our nurses get is very small. Nearly fifteen years ago I had almost completed a plan for the instruction of our Western Infirmary nurses in cookery, but difficulties arose, and some of our directors could not then see the importance of overcoming what seemed to me really very small obstacles. I hope the matter may now be taken up. Various methods could be adopted for dealing with it; but, in my opinion, the most satisfactory would be some arrangement by which those students who desired it, as well as the nurses, might receive on our own premises some practical instruction on this subject, which is so important to both.

### Wants and Workers.

ARRANGEMENTS have been made for the invalid for whom a home was wanted.—*Helpless.*

CAN anyone tell me if dispensing is taught to ladies anywhere in Manchester or Liverpool, and if there is any hospital where such instruction would be given to a trained nurse in return for her services?—*Sister Etta.*

### Nursing in Rio de Janeiro.

The Strangers' Hospital, which stands in the Rua de Passagem, Botafogo, is well known to those who have visited Rio de Janeiro, many travellers thankfully acknowledging the debt of gratitude they owe to the skilful care with which they have been nursed back to health. The outside of the building is considered somewhat difficult to photograph owing to its situation, but possibly the illustration of the interior on the next page may serve to give readers of The Hospital some idea of a place where English nurses have been doing very good work in a distant land. Fresh nurses have recently gone out to join the hospital staff, which for some months had dwindled down to three Englishwomen, one being the

Sister J. A. Jackson. Sister C. E. Bright.

English Pioneer Nurses in Brazil.

Superintendent, and the other two "Sisters." The work under such circumstances became both anxious and incessant, neither of the Sisters being able to take a holiday. Their labours during last year's epidemic of yellow fever, particulars of which appeared in these columns, followed closely on the experiences of another trying period, the revolution having naturally kept everyone in a state of nervous anticipation, accentuated by the shock of the constant firing. The two ladies, who alone of all those added at various times to the staff have been able to remain at the hospital, are Sister C. E. Bright, who was trained at the General Hospital, Wolverhampton, and was afterwards a ward sister at Prince Alfred's Hospital, Sydney, Lady Superintendent of the Children's Hospital, Brisbane, and Matron of the Croydon District Hospital in North Queensland; and Sister Jackson, who was trained at St. Bartholomew's Hospital, and after holding minor appointments became Superintendent of Nurses at Bradford Fever Hospital. With the advent of the new nurses sent out from England work and responsibilities will be lightened, and the present may therefore prove a brighter year than the last for the nursing staff at the Strangers' Hospital.

### Helps Homewards.

A year ago we quoted the words of a poor woman who described the untrained village attendant as a "easy goin' old creetur as knows nought 'cept how dying folks can be helped home quick and laid out 'ansome." We then hoped that the type thus aptly summarised was dying out, in favour of the skilled district nurse, but the helps homeward are still vigorous and within easy reach. We find them boldly combining "monthly" with "laying-out" work, and making personal profit at the expense of the helpless mothers and babes, who are equally ignorant of the risks they both run.

### The Lambeth Infirmary.

We insert with pleasure communications from correspondents who have personal knowledge of the valuable work done by the respected Matron of Lambeth Workhouse Infirmary during twenty-two years of office. For nearly six years previous to her appointment at Lambeth, Miss Griffiths worked under the Poor Law in Manchester, and much regret was expressed by the Board at her resignation. Before going there she had had some three years' experience in general hospital nursing.

In consequence of the improvements gradually introduced by Miss Griffiths at Lambeth, the work connected with the nursing department increased enormously, and two or three years ago the Local Government Board advised the addition of a second night superintendent, and a nurse already in the infirmary was selected by the Guardians to fill this post. The further recommendation of an assistant matron who should be also Superintendent of Nurses was likewise carried out, but the matron was not asked, as is customary in the best infirmaries, to make the selection. This appointment does not appear to have been advertised, but applications were invited from various hospitals, and Miss Tilbury obtained it by a majority of one vote. The title of Superintendent of Nurses given to the new officer caused unforeseen difficulties, and the Guardians, on being appealed to by Miss Griffiths last November on a question of discipline, resolved "that notwithstanding the appointment of Miss Tilbury, the Matron's authority as responsible head of the female staff is not to be interfered with." A recent application is reported to have been made by the Guardians to the Local Government Board for permission to abolish the office held by Miss Tilbury.

We have not yet heard the decision of the Local Government Board in the matter, but we hope it will secure the enforcement of discipline and restore complete harmony within the institution. This case is one more proof of the necessity for a revision of the rules governing the relations of medical superintendents, matrons, and other officers of Poor Law infirmaries to which we drew attention recently. It is not a question of fewer officers, for they are rarely sufficient for the work to be done at present, but of wiser regulations and better defined duties and responsibilities which must improve the whole administration of Poor Law infirmaries, and so promote the welfare and happiness of all concerned.

### Our American Letter.

(COMMUNICATED.)

ALTHOUGH we can well congratulate ourselves on the progress made in nursing of late years, and on the numerous excellent training schools which have been established, we find no small difficulty in getting accurate details of the work already accomplished in this country.

This is, of course, partly due to there being no published list of existing schools. Such a record would be of immense use to would-be graduates, who find it difficult to form approximately correct notions on the size and system of individual establishments. At present a "school" is a misleading title, being used indiscriminately to describe a community of four or of forty pupils.

Possibly some kind of record of all the larger training schools may be one result of the Convention of the American Society of Superintendents. At any rate, the want once acknowledged, it is probable that steps will be taken to supply it. The report of the next meeting of the Superintendents, which is fixed to take place next month in Boston, is awaited with interest.

There is some chance of a new establishment being erected in Minnesota which will be devoted exclusively to persons afflicted with epilepsy.

At Johns Hopkins Medical School ten out of the forty students who have entered there are women.

The Women's Hospital, New York City, has attained to its thirty-ninth anniversary, and a pleasant gathering of friends took place on the occasion. One of the wards was most tastefully adorned with rare plants and flowers, and was the scene of various addresses as well as a short service of prayer.

A new and larger building is desired for the Presbyterian Hospital and Free Clinic, which five years ago came into existence. The nursing staff consists of a matron and ten trained lady nurses.

Much active interest in hospitals is taken by women over here; for instance, at Washington 10,000 dols. were collected by them some time ago, to be applied to the erection of an infectious diseases hospital. Unhappily the site has not yet been secured, and there is actually no adequate provision for the cases of small-pox now in Washington. It is acknowledged with shame by the citizens that the present "Pest House" is most unsuitable for the purpose to which, failing better provision for infectious diseases, it has still to be put.

One of the first graduates of the Johns Hopkins Hospital Training School has been appointed Superintendent of the newly-established school at Baltimore, which is connected with the New Maryland Hospital. This lady, Miss T. Spencer, has been in charge of the Nursery and Children's Hospital at Baltimore for the last two years. Other recent appointments chronicled are as follows: Miss Nettie Romans to be Superintendent of the Hargons Hahnemann Memorial Hospital, Rochester, N.Y.; Miss E. Darling to be Superintendent of St. Luke's Hospital, Denver, Col.; and Miss S. C. Hearle to the Jefferson College Hospital, Philadelphia.

The Nurses' Home at the Toronto General Hospital was inspected by many visitors on the occasion of last year's graduating class receiving their badges and certificates. The pupils numbered twenty-one, and an entertainment of music, recitations, and addresses followed the ceremony. The school has been in existence about thirteen years.

At the meeting of the Board of Managers of the Hospital of the University of Penna, in December, it was decided that as soon as separate sleeping rooms could be provided, coloured applicants for training in the nurses' school would be accepted on the same conditions and terms as white. This is, so far as we know, the first occasion on which equal advantages have been offered to both races.

STRANGERS' HOSPITAL, RIO DE JANEIRO—LARGE GENERAL WARD, LOOKING WEST. *See page* cxx.

### The Muses' Looking Glass.

### THE NEW GALLERY.

### Exhibition of Venetian Art.

It is not long ago that Venice was represented at Olympia in a manner now familiar to us all. That was the Venice of amusement and frivolity. It is quite another Venice that we visit at the New Gallery—Venice of earnest hearts and busy hands. We are surrounded with relics from a past when art and work seemed synonymous terms within the beautiful sea-bound city. We cannot visit the New Gallery and not be struck with this characteristic. In this "made in Germany" age, with its tendency to cheapness rather than worth, it is a wholesome lesson, and one that provokes our admiration and our wonder, to study specimens of handicraft produced when men's work was part of themselves, and the eight hour question was unthought of.

The beautiful exhibition at the New Gallery consists of a very representative collection of pictures by Venetian artists from the 14th century upwards; of armour, bronzes, ivories, sculpture, medals, coins, furniture, glass, porcelain, autograph letters, jewellery, lace, and embroideries. As is always the case at the New Gallery, the exhibits are excellently and tastefully arranged and catalogued. The central hall is rendered picturesque by the quaint old well of carved red marble, with its bronze dipping ewer hanging from a supporting arch of metal work above. The gallery which runs round the hall is hung with beautiful tapestries, lending warmth to the sombre hues of the armour below. The general impression is one of a rich mosaic of colour, so typical of early Venetian taste. The pictures glow with that subdued sunlight which seems to have dominated the minds of all Venetian painters. In one picture where this tone is absent—an Assumption of the Virgin, by Giovanni Battista Tiepolo—it is as if an alien from Northern climes had found a resting-place in the sunny South, and the alien does not look by any means at home. The colouring is clear, bright, and cold, and the drapery altogether lighter and more fantastic than in any other picture present. Out of the many beautiful examples of Venetian pictorial art, it is difficult to choose any one for remark more than any other. Amongst the Holy families which form the major part of the collection, "A Virgin and Child," by Giovanni Bellini, is, perhaps, one of the most charming. The face of Mother and Child are both so serenely sweet and pleasing. The general characteristic of the surrounding faces is a little sad and severe. Then there is a portrait of one of the Doges, "Antonio Grimani," by Titian, which must arrest attention. The cynical penetrating glance is vivid and lifelike; the firm, pitiless expression so haunting, that we feel we stand before one of the painter's masterpieces. The Holy Family, by Bonafazio (No. 89), contains a beautiful representation of typical Venetian beauty in the Magdalen. She is portrayed with the glorious red bronze hair, dear to the painter's heart, and to this the artist has added a lovely face of perfect feature and colouring. Besides the paintings which come from many celebrated private collections, there are a number of interesting drawings, some of which have been lent by her Majesty the Queen.

In the gallery the autograph letters form a most attractive part of the collection. Here we can read the beautifully-formed characters of the great Titian, whose handwriting was worthy of the skilled hand that guided the pen. Very little less a work of art is the handwriting of Torquato Tasso, the poet, who writes for that Renée of Farrara (to whom he was secretary) rendered of mortal interest through Schiller's work. Renée's signature is attached to the letter. There are also interesting letters from Paul Veronese and other names celebrated and familiar.

Beautiful indeed are the bookbinding, the lace, and the embroideries. Amongst the lace, a most quaint specimen representing the history of St. John the Baptist should not be overlooked. The work is wonderful, but the figures are grotesque in the extreme.

Some of the inlaid armour is well worth attention, and in case M are shown some exquisite glass goblets. There were better examples of Venetian glass, however, at the exhibition of early Italian art. Finally, it is needless to say the exhibition is worth visiting. The name of Venice carries a charm of its own, and as in the present case the best products of its art have been brought together, no further inducement is needed. There is a quiet and restfulness too, peculiar to the New Gallery, which adds to the attraction of its exhibitions, and causes a visit thither to be a refreshing experience rather than a fatigue.

### Where to Go.

St. Martin's Town Hall.—The Queen has graciously consented to be patron of a bazaar to be held in aid of the Royal Westminster Ophthalmic Hospital. It will be opened on May 14th by H.R.H. the Princess Henry of Battenburg.

St. John Ambulance Association.—A meeting will be held at Fishmongers' Hall, E.C., on Monday, 21st inst., at five p.m., presided over by Sir Albert K. Rollit, M.P., to consider the question of ambulance training or board ships amongst officers, crews, passengers, and emigrants. The meeting has been organised by Captain A. G. Froud, R N.R., secretary of the Shipmasters' Society, and Mr. Alan Palmer, hon. secretary for the City and Port of London St. John Ambulance Association.

East London Hospital for Children.—The entertainment for the nursing staff will be held at eight p.m. on January 26th.

### Presentations.

On Monday, 7th inst., a handsome cake basket was presented to Dr. Keats, at Greenwich Union Workhouse, by the Matron, Mrs. Carter, on behalf of the nurses, in token of their appreciation of the course of lectures given by him at the infirmary.

### Notes and Queries.

The contents of the Editor's Letter-box have now reached such unwieldy proportions that it has become necessary to establish a hard and fast rule regarding Answers to Correspondents. In future, all questions requiring replies will continue to be answered in this column without any fee. If an answer is required by letter, a fee of half-a-crown must be enclosed with the note containing the enquiry. We are always pleased to help our numerous correspondents to the fullest extent, and we can trust them to sympathise in the overwhelming amount of writing which makes the new rules a necessity. Every communication must be accompanied by the writer's name and address, otherwise it will receive no attention.

### Queries.

(59) *Incurable.*—Where can I get information about Homes for Incurables?—*L. N.*

(60) *Packs.*—Kindly inform me what book gives a good description of hot and cold packs?—*Nurse C.*

(61) *Matron's Holiday.*—Please tell me if the matron of a cottage hospital must provide her own substitute during her annual holiday, and if a month is too long for her to expect?—*Entre nous.*

(62) *Ambulance.*—To whom should I apply for particulars as to St. John's Ambulance classes?—*Matron.*

### Answers.

(59) *Incurable* (*L. N.*)—See "Burdett's Annual," published by the Scientific Press, 428, Strand.

(60) *Packs* (*Nurse C.*).—"Lectures on Nursing," by Eva C. E. Lückes, contains excellent directions for these.

(61) *Matron's Holiday* (*Entre Nous*).—If the matron has no nurse friend who can take her place during her holiday, the committee would have little difficulty in finding a trained nurse glad to have the useful experience of such a charge. A month is certainly not too long.

(62) *Ambulance* (*Matron*).—To the Secretary, St. John's Gate, Clerkenwell, London.

### Nursing in Dublin.

SIR PATRICK DUN'S HOSPITAL.

This hospital holds a deservedly high position, and it is to be hoped that the contemplated effort to improve its finances by a fête in the spring may be successful.

It must not be imagined that the debt in which the hospital is at present involved is the result of anything save depreciation in the value of landed property. By this the hospital has lost an income of about £1,000 a year.

Sir Patrick Dun was a distinguished member of the medical profession, and by birth a Scotchman. He flourished in the reign of "the glorious, pious, and immortal King William," and in the capacity of "physician to the army" he is said to have dressed the wound received by that monarch at the Battle of the Boyne. He was President of the Irish College of Physicians, sat as member for Mullingar in the Irish Parliament, and died bequeathing his considerable property to found and endow a professorship of physics. This bequest in process of time becoming much more valuable, it was determined in 1800 to devote a large portion of the money to establishing a hospital in Dublin with the aid of a grant from Parliament.

In outward appearance this hospital is a substantial building of grey stone, the main portion probably dating from the beginning or middle of the last century. Various additions have been made, such as the out-patients' department, the operation theatre, and the new fever wing; the latter, a detached building, superseding the old fever wing, which, forming part of the main building, was unsuitable for infectious diseases. This is now utilised for maternity wards, private patients, &c.

In the older portion of the hospital the floors of corridors, staircases, and wards are chiefly of stone, with pretty rugs and mats, polished tables, the latter with basin and ewer, writing materials, and vases of flowers. The walls of one ward have been effectively frescoed, under the auspices of the Kyrle Society, by well-known Dublin artists.

A hitherto disused apartment on the ground floor is to be converted into a day-room for men, which will no doubt be appreciated. The hospital affords accommodation to one hundred patients. As a training school for nurses and midwives it stands well, and the marked improvement manifested in private and hospital nursing reflects great credit on the lady superintendent.

Probationers on entering the hospital are required to pay an entrance fee, and are only admitted on the distinct understanding that they mean to remain for three months although if considered unsuitable by the lady superintendent they may be dismissed at the end of one. Supposing, however, they are desirous of remaining in the service of the hospital and the lady superintendent is willing that they should do so, at the expiration of three months, they are paid at the rate of £10 the first year, increasing by £2 during each subsequent year of their training. It is expected that in the course of eighteen months they will become good nurses, but their engagement requires them to remain in the service of the hospital for two years and a-half longer in hospital, district, or private work according as the lady superintendent may decide.

A six months course of practical instruction in midwifery under the superintendence of the King's Professor may be secured by payment of a fee of £5 in connexion with the maternity department of Sir Patrict Dun's Hospital, furnished apartments, fuel, and gas being provided by the board for those undergoing instruction. A diploma is conferred by the governors of the hospital on the completion of the specified course.

### Entertainments.

On Thursday evening, 9th inst., the matron of Guy's Hospital held an "at home" in the fine old Governors' hall of that institution. A large gathering of the nurses and their friends took place, the treasurer and many members of the medical staff and school being also present. The hall was tastefully arranged as a reception-room, fine palms and standard lamps having a charming effect in the spacious apartment. Excellent music and other entertainments filled up a very agreeable evening.

Christmas diversions were brought to a close at the Home and Hospital for Incurable Children, 2, Maida Vale, on Friday, 11th inst., by a Christmas tree, kindly provided by Mrs. Soames and the Misses Yolland. The little patients thoroughly enjoyed the spectacle, from the lighting of the first taper to the extinction of the last, and the distribution of toys was eagerly welcomed by the happy, well-cared-for little creatures to whom at such a moment the word "incurables" seemed altogether inappropriate.

A concert in aid of the funds of the Metropolitan Hospital, Kingsland Road, was given at Shoreditch Town Hall on the 10th inst., and was well attended.

An enjoyable entertainment was given to the children in the infirmary at the South Metropolitan District Schools on December 28th. It was arranged by Miss Thornton, the matron, with the assistance of her nurses, and their combined efforts secured a most amusing programme.

The in-patients of the Cancer Hospital, Brompton, had a pleasant entertainment on the 8th inst. when an excellent programme of music, tableaux, and recitations was arranged for them by Miss Constance Marsden and other friends. The hospital was very prettily decorated by Matron and Nurses.

At Sheffield General Infirmary a very pleasant Christmas was spent by the patients and staff. The wards and chapel were beautifully decorated, and in the latter a bright and cheerful service was held, at which all who cloud were present. Carol singing and Christmas fare marked the day, and an excellent display of tableaux vivants took place in the evening. On December 27th the usual Christmas tree was provided for the younger patients, and the following evening an entertainment took place in the new building. The festivities concluded with the boys' annual entertainment on New Year's Eve, when Father Christmas presided at a well-furnished tree.

Christmas was observed with due honour at the York County Hospital, the festivities beginning on Christmas Eve, when the sisters and nurses sang carols in the wards. At noon the patients dined sumptuously on turkeys (provided by the liberality of a private patient), plum pudding, and dessert. In the evening a musical entertainment was given by the chaplain and resident staff. On the 27th the Christmas tree was held, preceded by an excellent tea, supplied by generous friends of the hospital. The Very Rev. the Dean of York (chairman of committee), assisted by Lady Emma Purey Cust, the matron, house surgeons, chaplain, and visitors, distributed the presents, each patient receiving two garments as well as a small gift. Musical and dramatic entertainments took place in each ward; and on January 1st tableaux and a Christy Minstrel troupe afforded much amusement.

On Tuesday, January 8th, an entertainment was given by the governors and medical staff of Dr. Steeven's Hospital, Dublin, consisting of a concert and dance, for which a large company assembled. The large hall in the left wing of the hospital was utilised, and the refreshments were served in the picturesque board-room, the corridors being lit up by hundreds of fairy lamps. Fine palms were kindly sent for the occasion from the Viceregal Lodge, and also stage properties, Royal Standards, and other decorations. On the following evening members of the staff and their friends gave a concert for the amusement of the patients, in whom they found a highly appreciative audience.

### Everybody's Opinion.

[Correspondence on all subjects is invited, but we cannot in any way be responsible for the opinions expressed by our correspondents. No communications can be entertained if the name and address of the correspondent is not given, or unless one side of the paper only be written on.]

### TRAINING AT LAMBETH WORKHOUSE INFIRMARY.

"Nurse Superintendent" writes: In the notice of Lambeth Infirmary in the "Nursing Mirror" of January 12th I regret to see that no mention is made of Miss Griffiths, the matron, from whose valuable services I personally have reaped advantage. The spirit which Miss Griffiths has succeeded in inspiring in many nurses whom she has trained can only be realised by those who have employed them as I have done. I do not think it is encouraging for other infirmary matrons to have the services of so valuable a worker entirely passed over in a notice of any particular infirmary. Who but the matron can inspire nurses in the same degree with a love of their work and a spirit of devotion to the sick poor? These qualities I have found especially marked in nurses trained under Miss Griffiths whom I have employed during the past ten years, and long before a trained assistant matron was appointed at Lambeth Infirmary.

"Perplexed" writes: I feel puzzled by an interesting little account in last week's Hospital of the training of probationers at Lambeth Infirmary, and hope for more information respecting the matter. Your correspondent writes as if training nurses was a new departure at Lambeth, whereas I and many others have had long experience of excellent nurses which have been trained there under Miss Griffiths, the matron. This lady is not mentioned at all, yet she is the person responsible for the training of the nurses, and to whom I have always referred regarding them. If this recent arrangement has been so successful, why do the Guardians wish to abolish the post of assistant matron? I saw it stated in a Lambeth paper a few weeks ago that the new assistant matron had caused great friction in the infirmary, two parties being formed which was naturally subversive of all discipline. Surely there must be some mistake, for it seems unlikely that an assistant matron would be appointed to an infirmary if she were the sister-in-law of the medical superintendent, as Miss Tilbury is said to be. Altogether I feel puzzled, and shall hope to see some light thrown on the subject shortly. Everyone who has had anything to do with poor law matters is well aware of the great difficulties which can be thrown in the way of a matron by the medical superintendent with regard to the female staff. Lambeth is doubtless another example of the desirability of speedy alteration in the Local Government Board orders with regard to the relative positions of the matron and medical superintendent.

### THE LONDON ASSOCIATION OF NURSES.

Miss Emma Durham writes: The first time I saw Miss Firth, the founder of this Association, was on my return to England from Zanzibar, whither I had gone, after my three years' training at St. John's House and King's College Hospital, to help start the first hospital in Zanzibar. I felt so strong in 1875 when I started that it seemed impossible that any climate could affect me. I returned to England in 1877 to look for other work, and my sisters fitted me out with warm clothing (for though it was July, and others felt the heat, I was shivering). After a few days in London I counted up the cost; I found I was already £12 in their debt. "Never mind," said my sisters, "don't bother about that!" Then I considered. If I went back to the nursing I had been at before going to Zanzibar, £22 a year and uniform would be mine, with board and lodging when not at a case. It would then take me more than six months to pay my debt, and I wanted to earn the money much sooner. For this reason I sought out Miss Firth, who had in 1873 formed the London Association of Nurses. Miss Firth had heard of me through one of my sisters, who had joined the Association in 1874, and received me very kindly, and after a chat she looked through my testimonials, and, just when I expected her to say "I will take you at once," she got up and, putting her hand on my shoulder, said, with a kindly smile, "You must go home and rest and feed up, live in the fresh air, and when you are strong I will find you some work to do." "Could I not work now?" I asked, for I had had more than a month's rest already coming home, and I had been told that at this Association I should earn from £60 to £80 a year, so I felt anxious to begin. Miss Firth only shook her head, and said I must get stronger, so I went home to my mother, and in September came back to town and joined the staff of the London Association of Nurses. For some time I still suffered from the effects of African fever and had to leave a few cases, but in spite of this by March 25th, 1878, I had paid my bills, "For I would rather go supperless to my bed than rise in debt in the morning." I wish I could tell my fellow-nurses what Miss Firth was—I mean those of you who did not know her. When no one else seemed to have given a thought to the difference between a nurse's earnings and her receivings, she struck out boldly for the betterment of the position of trained nurses. There was no sounding brass or tinkling cymbal, it was done quietly and silently as most good work is. Many remember Miss Firth with gratitude, as I do, for it was not only in our nursing work that her counsel and judgment were so valuable, but her large-heartedness and broad mindedness and ready sympathy with all women with whom she came in contact, were an example to all. Who that remembers Miss Firth's lectures (before lectures were generally given in nursing homes) will ever forget her wise and loving advice to us to imitate many who had gone before, and then if it were our vocation we should prove true nurses for the sick and suffering.

### SPURIOUS CO-OPERATIONS AND HOMES.

"A Well-wisher of 'The Hospital'" writes: I see in last week's issue that you very properly warn nurses against joining a co-operation in a northern town. But allow me to tell you that there are several so-called co-operations which are private adventures of persons who are quite out of sympathy with nurses. These become delusions and snares to many honest, hard-working nurses in search of employment. In some a fee, ranging up to a guinea, is extracted from the applicant before a "case" is guaranteed, and a heavy commission on every guinea earned is charged in addition. At the end she will probably be told by the matron that she is no longer required. Again, there is a "co-operation" where for the first three months 5s. in the guinea is charged as commission, the nurse often being put to the trouble of finding her own work through doctors of her acquaintance. At the end of the three months she has to pay a guinea subscription, and afterwards 2s. 6d. in the guinea on her earnings. The co-operation in question, like many of its kind, has very little work to give, and depends chiefly on the nurses finding their own cases. During the so-called "slack time," which lasts for some months of the year, the nurses crowd miserably together in the "Home," many, if they can, going away to their own people, whilst waiting for work. I have known nurses in the home pay their board with borrowed money, and then probably leave in debt in the end. When a few cases do come in, nurses are advertised for, instead of the applications being sent on to other institutions, as the better-known associations do. The new nurses, often fresh from hospital, get treated in due time in the same way as the former ones, and they, too, disappear in their turn. The Home itself is often dirty and comfortless, though pretentious, and the food frequently is of the worst quality, wretchedly cooked and served. A nurse may be told on arriving back from a case (after paying her commission) that there is no bed for her, and if she has no home in London she must wander forth to look for a lodging. I would warn all nurses against co-operations of the kind. If they cannot get on the staff of a good co-operation with a committee, or join a reliable institution, let them stay in a hospital, where they will gain experience, be looked after in sickness, and be in the end far better off in health and pocket than in any of these places of mushroom growth.

## Figures and Tables

**Fig. 1. f1:**
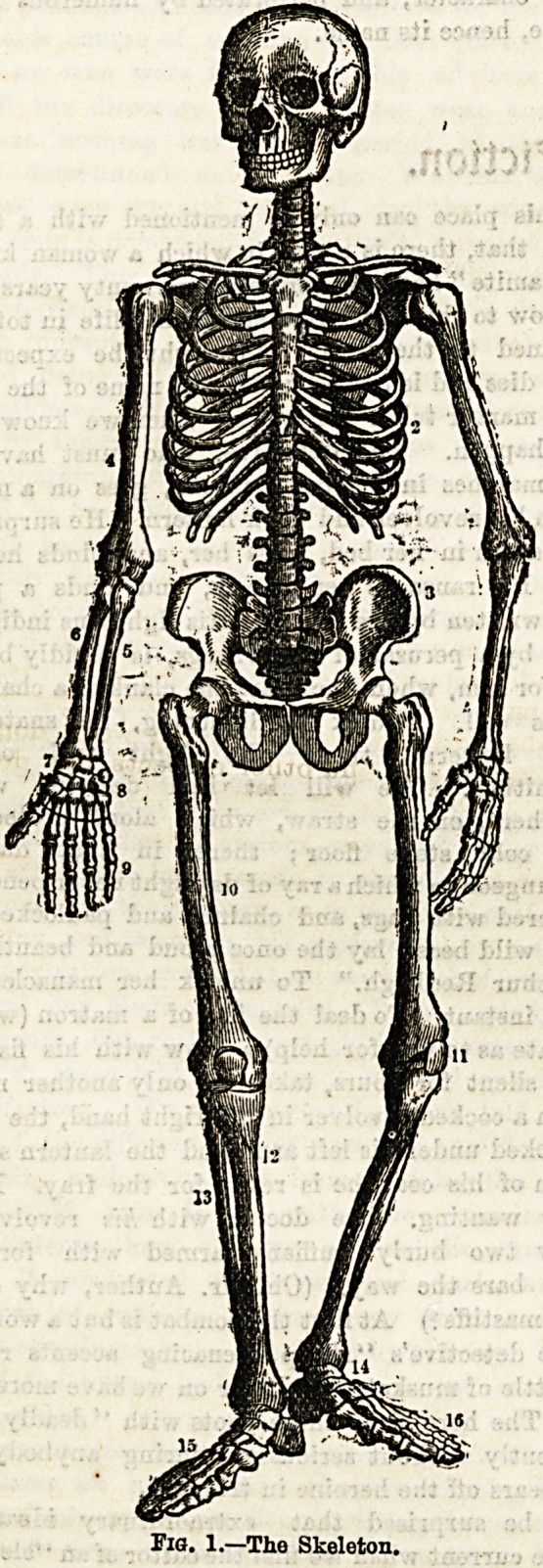


**Figure f2:**
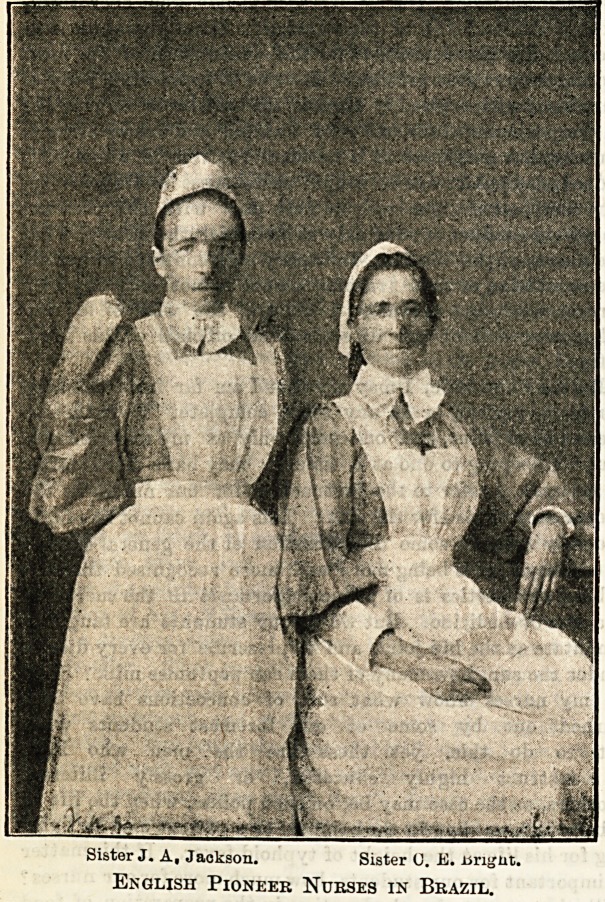


**Figure f3:**